# Reducing the probability of false positive research findings by pre-publication validation – Experience with a large multiple sclerosis database

**DOI:** 10.1186/1471-2288-8-18

**Published:** 2008-04-10

**Authors:** Martin Daumer, Ulrike Held, Katja Ickstadt, Moritz Heinz, Siegfried Schach, George Ebers

**Affiliations:** 1 Sylvia Lawry Centre for MS Research, Hohenlindener Str. 1, 81677 Munich, Germany; 2 Department of Statistics, University of Dortmund, 44221 Dortmund, Germany; 3 Institute for Medical Biometry, Epidemiology and Computer Science, Clinic of Gutenberg's University Mainz, Germany; 4 Department of Statistics, University of Dortmund, 44221 Dortmund, Germany; 5 University Dept of Clinical Neurology, Oxford University, UK

## Abstract

**Background:**

Published false positive research findings are a major problem in the process of scientific discovery. There is a high rate of lack of replication of results in clinical research in general, multiple sclerosis research being no exception. Our aim was to develop and implement a policy that reduces the probability of publishing false positive research findings.

We have assessed the utility to work with a pre-publication validation policy after several years of research in the context of a large multiple sclerosis database.

**Methods:**

The large database of the Sylvia Lawry Centre for Multiple Sclerosis Research was split in two parts: one for hypothesis generation and a validation part for confirmation of selected results. We present case studies from 5 finalized projects that have used the validation policy and results from a simulation study.

**Results:**

In one project, the "relapse and disability" project as described in section II (example 3), findings could not be confirmed in the validation part of the database. The simulation study showed that the percentage of false positive findings can exceed 20% depending on variable selection.

**Conclusion:**

We conclude that the validation policy has prevented the publication of at least one research finding that could not be validated in an independent data set (and probably would have been a "true" false-positive finding) over the past three years, and has led to improved data analysis, statistical programming, and selection of hypotheses. The advantages outweigh the lost statistical power inherent in the process.

## Background

The validity of published research findings is receiving appropriate scrutiny [1-4]. Erroneous conclusions are commonplace [1-4]. Analyses performed on datasets prior to focus on specific hypotheses or models do hazard the generation of hypotheses filtered through unwitting bias. Hypothesis-generating experiments are necessary but multiple model selection may not be capable of identifying valid conclusions. Pre-publication validation aims to reduce the number of false positive findings.

Multiple sclerosis is a disease of the nervous system with highly variable outcomes. Relapses are characteristic and average 0.5/year [5] in the relapsing phase. Half of patients need aid for walking or are worse after 15 years [6]. In clinical trials annualized relapse rates and disease progression (as measured by the Expanded Disability Status Scale or EDSS) have been used as endpoints. Magnetic resonance imaging (MRI) of the brain detects inflammatory activity and change in brain volume. MRI-related endpoints include new gadolinium-enhancing lesions and total brain lesion volume (T2 weighted image) but remain unvalidated surrogates for long-term outcome.

Several medications reduce relapse rate and/or MRI lesions but are uncertain suppressors of disease progression. The Sylvia Lawry Centre for MS Research (SLC) was developed to improve outcome-based trial research in MS. We describe the background of the SLC, its framework for statistical validation and studies demonstrating the Centre's policies.

### The validation policy of the Sylvia Lawry Centre for MS Research

The validation policy of the SLCMSR prescribes a random split into *two *parts for hypothesis generation and validation, a variant of independent replication by split sample validation.

Training and validation parts contain 40% and 50% of the data respectively. When new databases are added, the remaining 10% of data is used for mixing purposes. The training part is available to researchers/statisticians for exploration and investigation and important findings are selected for validation. Approved proposals go to the "data-trustee", who evaluates the validation dataset, and summarizes the result for the publication/validation committee, the analyst and collaborative researchers. Results obtained from the training part of the database, annotated by confirmation information are published (see Additional file 1, resp. Lit. [7]).

Proactive application of this process applied to SLC projects coming to the final validation step is described [8-11]. A common goal in chronic diseases is to find predictors of an outcome variable of interest among larger sets of potential explanatory variables. These variables can be continuous, binary or count variables and lead in turn to linear, logistic, or Poisson regression models. After identification of significant predictors in the training portion of the database in a multiple regression model, we attempted to confirm these in the validation part of the database. By "successful validation" we meant that the same predictors remained significant on a 5% level in a multiple regression model in the closed part (see, e.g., Altman).

A simulation study assessed the influence of variable selection on the overall significance level. In practice, the distribution of key variables and number of patients in the specific subgroups are determined within the validation step to ensure correspondence of the datasets.

## Methods

### a) Case studies and examples

#### Example 1

**Figure 1:**
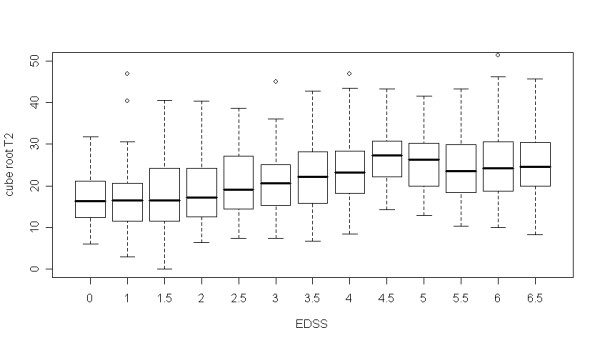
Plateauing relationship between EDSS and T2 lesion volume in the open part of the SLC database [8].

Relevant to the use of T2 lesion volume as a surrogate marker for disability, we investigated the relationship between this MRI outcome variable, and a set of continuous, ordinal and binary clinical determinants as potential explanatory variables. Unexpectedly, a non-linear plateauing relationship between the ordinal predictor Expanded Disability Status Scale (EDSS), and the MRI outcome for a set of 1312 placebo patients with MS from randomized clinical trials was found (Fig. 1, see [8] for details on the variables and results of this project).

We validated this finding in steps, the key step being whether the EDSS predictor led to improvement (p < 0.05) of model fit when entered in a nonlinear (as compared to linear) fashion. We then calculated Spearman's correlation coefficient over the range of EDSS values and considered the validation of the plateauing relationship successful, if the overall correlation with EDSS was positive and significant (p < 0.05), and if the correlation coefficient for EDSS >4 was not significantly different from zero, (95% confidence interval included zero). The size of the validation sample corresponding to this project contained 848 patients. The distributions with respect to the key variables were similar in training and validation sets, indicating comparability and suitability for validation. All predictors assessed were validated in the multiple regression fits. The major finding of a plateauing relationship between EDSS and transformed T2 lesion burden was unequivocally confirmed – T2 lesion volume does not seem to be a good surrogate for disability in MS patients.

#### Example 2

The development of gadolinium enhancing lesions is often used in phase II clinical MS trials to evaluate the potential efficacy of new drugs. The presence of these lesions is interpreted as an indicator of acute disease activity in MS patients [9]. Predictors of enhancement status could be useful for the selection of patients for MRI monitored trials.

In a multiple regression model with clinical and demographic predictors ("disease course", "age at disease onset" and "disease duration" [9], the MRI predictor T2 lesion burden significantly improved the prediction of enhancement status in the open part of the SLC database. We then defined the validation to be successful if the values of several statistics in the closed part of the database, most importantly the positive predictive value, are above the lower endpoints of approximate one-sided 95% prediction (99% for "excellent" validation) intervals for the anticipated value in the validation part of the database. We found that the increase in positive predictive value over the a priori chance of enhancement in the closed database when T2 lesion burden was included as predictor exceeded the prespecified level defining "excellent" validation.

#### Example 3

We investigated within trials whether relapses contribute to the development of subsequent sustained increase of impairment and disability in patients with MS as measured by the EDSS [10]. On-study relapse data was collected in so-called "sacrifice" periods of 80, 120, 160, or 200 days. Confirmed increase in EDSS was defined as at least one point rise confirmed by another visit at least 135 days later. In two comparison groups with two different cut-point splits: a) 0 versus at least 1 relapse during the sacrifice period, and b) 0 or 1 relapse versus at least 2 relapses during the sacrifice period, analysis was based on a two-sided log rank test to determine whether time to confirmed rise in EDSS is the same for two groups. Results are displayed in terms of hazard ratio and 95% confidence interval. There were 256 relapsing remitting MS patients in the training database for this analysis. Combining the four different sacrifice periods, and the two different cutpoint splits results in eight tests. The test with the smallest p-value was the one for 120 days sacrifice period and cut-point split 0 versus at least 1 relapse during sacrifice period (likelihood ratio test p-value was 0.0012, estimated hazard ratio = 2.26, 95% confidence interval [1.36; 3.75]). Such a result if validated – would support the assumption that reduction of relapses slows down the accumulation of disability.

In the 320 patients available in the validation sample comparable to the training sample for distribution of the key variables (p-value of the one-sided Wald test was 0.109) findings could not be validated. We concluded that "there is no consistent effect of on-study relapses on the subsequent development of sustained EDSS score increase during a typical clinical study observation period".

### b) Simulation studies and validation cost

In simulation studies to determine the effect of variable selection on the significance level of global F-tests in multiple regression analysis (data not shown), observed error rates were found to exceed 20% for both forward and backward selection. With only one or two variables in the model, forward selection has more flexibility to identify "significant" predictors, leading to more false positive findings than backward selection. Forward selection and backward selection produce comparable error rates with 3 or more variables. With six predictors in the regression model, forward selection maintains the significance level of 5%.

**Figure 2:**
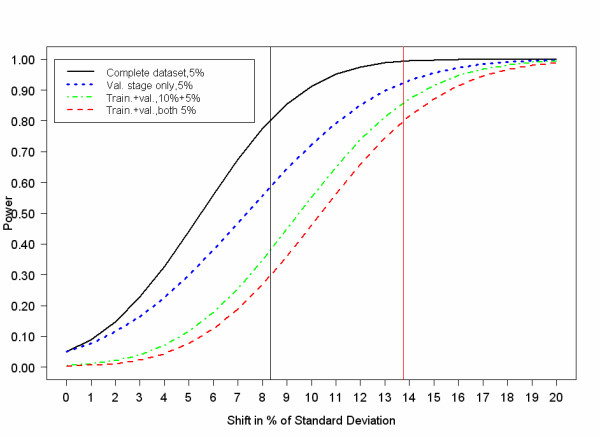
Displays the power for four different validation scenarios plotted against shift in percent of standard deviation: the first for the complete dataset on a 5% significance level; the second for testing on the validation stage only (N = 500 patients) on 5% significance level; the third for testing on the training set (N = 400 patients) with 10% and on the validation set (N = 500) with 5%; and the fourth scenario for testing on a 5% significance level in the training data (N = 400 patients) and in the validation data (N = 500 patients). There is a sacrifice in power using this validation scheme.

To assess the "cost" of splitting the database we used a one-sided, one-sample Gauss-test situation with 900 observations, (400 would be available in the training database, and 500 for confirmation; see Figure 2).

## Results

Since the SLCMSR validation policy was put in place, ten larger scientific projects have been finalized using the training portion of the database. In five of them, the findings of the training data were selected for confirmation in the validation part of the database. In four of these, training dataset findings were replicated in the validation dataset. In one, the "relapse and disability" project as described in section II (example 3), findings could not be confirmed in the closed portion of the database – without the validation policy this would have lead to publication of a false-positive finding. The other five projects were finalized without result validation. In each case the authors, committee members and journal reviewers agreed that the findings obtained in the training set were sufficient for answering the question at hand. In some cases the hypothesis had clearly been formulated before touching the training set. In another example only a rough estimate of the upper bound of the mortality rate was proposed to decide about the feasibility of a trial design [12,13].

## Discussion

Exploratory data analysis typically starts with data description entailing comparisons, and often generating statistical hypotheses. Ideally, however, all hypotheses should be formulated *prior *to descriptive analysis (or even before data collection). In practice data description leads to new questions, to investigation of new relationships by formulating hypotheses, and then formal testing. Descriptive statistical analyses can substantially endanger the validity of formal statistical inference by destroying the probabilistic basis of inferential statistics.

Substantial statistical methodology has been dedicated to overcoming this problem including replication, cross-validation, limits for family-wise error rates and Bonferroni-adjustment for multiple testing [14,15]. These methods could be applied to control the overall significance level for a type I error, but it is usually impossible to quantitate prior "data dredging" [15-19].

Significance levels can be controlled by dividing the data set into separate parts prior to data analysis. Hastie et al. [16], for example, recommend randomly splitting the database into 1) a training set, 2) a validation set, and 3) a test set to evaluate the predictive accuracy of the model. Van Houwelingen and le Cessie [20] suggest using one part of the database to select the covariates, a second to estimate the regression coefficients, and a third to assess the prediction rule.

The SLCMSR hosts a large database on multiple sclerosis patients from clinical trials and natural history studies. Data donors do not influence the publication process, and SLCMSR follows strict rules guaranteeing non-identifiability of individual data sets. Anonymization and splitting of the trials make it impossible for SLCMSR and collaborative researchers to identify patients and trials with individual data donors.

Splitting the large SLC database into two parts yields one training part for hypothesis generation, and a second for validation purposes. Only large databases are suitable for splitting, because in secondary analyses patient numbers drop considerably. The validation part of the database is reserved for confirmation of single pre-specified hypotheses. The major finding of one otherwise finalized project could not be validated, and the publication of a false-positive finding was prevented. More recent findings [21] suggest that even a consistent effect of on-study relapses on subsequent "sustained progression" could not be interpreted as evidence for a link between relapse frequency and the accumulation of true, unremitting disability.

However, one should be aware that it can of course not be excluded that in some cases the application of the validation policy will lead to the publication of false negative findings. This disadvantage is related to the inherent loss of power induced by the split of the data base. Therefore, we think that our procedure is particularly useful in areas where the presence of false-positive findings and a considerable degree of "optimism" is common. This is certainly true in MS research, but may be frequent in other areas of clinical research as well.

Moreover, we believe that in general having this validation policy leads to a more sensible and thorough data analysis, programming and code checking, and selection of hypotheses to validate.

Simulations of the true significance level under the null hypothesis of global F-tests after forward and backward variable selection showed (N = 746) that the significance level can easily go beyond 20% when only a small number of predictor variables are included in the model.

The price to pay for splitting the database is a reduction in statistical power. We simulated power levels similar to a typical study at the SLC, and we demonstrated that the shift in percent of standard deviation for a one-sided Gauss-test detected with 80% power needs to be nearly double the size with result validation than without result validation. In other words, statistically significant findings need to be detected twice: in the training sample and in the validation sample. However, the price of publishing false positive research findings in a field with many false dawns justifies validation efforts.

Is the proposed method of result validation generally suitable for research questions or databases? We think that properly designed randomized controlled clinical trials do not necessarily need result validation – although completely separate and independent replications should not be discouraged. Even when additional hypotheses are to be tested at the end of the trial, Bonferroni adjustments can be sufficient to control the significance level. Epidemiological studies, however, are not scientific experiments, and, the study design is less structured than in clinical trials, and often lacking randomization.

In addition false-positive findings from large-scale studies cannot be disproved since other studies are typically smaller and do not have the power to do so. When a large group of researchers works on a scientific field using the same database, result validation is a powerful way to reduce the probability of publishing false positive findings.

Allision [22] states that there is an ongoing debate whether studies with microarrays require any validation guidelines that are fundamentally different from other types of study [23,24].

We think that different ways to construct the open and closed part of the data base – e.g. selecting randomly individual data packages (or random fractions thereof) to go either in the open or in the closed part – may be interesting modifications of our policy. Future work will aim to define the exact goals of such extended validation studies and at an assessment of which validation procedures will meet those goals.

Ioannidis [1] states that there is no "gold-standard" for validation in general, but that the percentage of published false positive findings can be reduced by better-powered studies, i.e. large-scale studies, low-bias meta-analyses, registration of studies and networking of data collections – similar to randomized controlled trials, and a split-team approach.

## Conclusion

We conclude that the validation policy has prevented the publication of at least one research finding that could not be validated in an independent data set (and probably would have been a "true" false-positive finding) over the past three years, and has led to improved data analysis, statistical programming, and selection of hypotheses. The advantages outweigh the lost statistical power inherent in the process.

## Competing interests

The author(s) declare that they have no competing interests.

## Authors' contributions

MD is director of the Sylvia Lawry Centre (SLC) and co-author of the original SLC validation policy (see also additional file to this manuscript). He has collected the data and supervised the research projects conducted at the SLC, developed the idea for this manuscript, wrote the first draft and prepared the various revisions. UH has participated in several research projects at the SLC and helped in writing the manuscript. MH finished his diploma thesis at the SLC on the topic "Validation Processes of Results in Biomedical Research Centres" and provided valuable input to the manuscript together with his supervisor KI, in particular about the simulation study. SS is co-author of the SLC validation policy, was involved in several research projects at the SLC and has helped to write the manuscript. GE is member of the Scientific Advisory Committee and the Multiple Sclerosis Expert Panel of the SLC. He worked in several research projects at the SLC and helped in writing and critically reviewing the manuscript. All authors read and approved the final manuscript.

## Pre-publication history

The pre-publication history for this paper can be accessed here:



## Supplementary Material

Additional file 1 Validation policy of the Sylvia Lawry Centre for Multiple Sclerosis Research entitled "Maintaining high quality of statistical evaluations based on the SLCMSR database. Validation Procedure of the SLCMSR" by Schach S, Daumer M, Neiss A (2003). This paper provides the basis for this manuscript and is also available at the homepage of the SLCMSR . Click here for file
