# Supplementary material for: Reducing the probability of false positive research findings by pre-publication validation – Experience with a large multiple sclerosis database
Source: BMC Med Res Methodol. 2008 Apr 10;8:18. doi: 10.1186/1471-2288-8-18 (PMC2358908; doi:10.1186/1471-2288-8-18)
Supplement: Additional file 1 — Validation policy of the Sylvia Lawry Centre for Multiple Sclerosis Research entitled "Maintaining high quality of statistical evaluations based on the SLCMSR database. Validation Procedure of the SLCMSR" by Schach S, Daumer M, Neiss A (2003). This paper provides the basis for this manuscript and is also available at the homepage of the SLCMSR . [file 1471-2288-8-18-S1.pdf]

SLCMSR Sylvia Lawry Centre for Multiple Sclerosis Research

Validation Policy

Maintaining high quality of statistical evaluations based on the SLCMSR data base

Prof. Dr. Siegfried Schach, Dr. Martin Daumer, Prof. Dr. Albrecht Neiß

Sylvia Lawry Centre for Multiple Sclerosis Research
Hohenlindener Str. 1
D-81677 Muenchen

Tel.: +49 (0) 89 2060 26950
Fax: + 49 (0) 89 2060 26951
Internet: www.slcmsr.org

Maintaining high quality of statistical evaluations based on the SLCMSR data base.

S. Schach, M. Daumer, A. Neiss

09.07.2003

1. Summary

For the purpose of maintaining highest standards in the statistical evaluations based on the SLCMSR data base, the following procedure is recommended:

The pool of data consisting of the collection of every study presently included or to be included in the future to the database should be split at random into an 'open' part and a 'closed' part, both of which have approximately the same size. Only a small group of “data trustees” shall have access to the closed part.

Researchers working with the data base will only have access to the open part of the data base. They may use this part for modelling purposes, generation of hypotheses and confirmation of hypotheses. Following internal approval a researcher’s request for validation will be decided upon by a validation committee with a turn around time of two weeks. The researchers can then validate their findings before publication by submitting to the trustees their methods and their program code. The data trustees use this information for computing the relevant results on the basis of the closed part of the data set. In their publication the researchers are then expected to amend their findings derived from the open data set by information about the outcome of the validation step in the closed data set.

In order to keep the two data sets as comparable as possible, the data trustees are obliged to make any changes to the closed part of the data set that have been made to the open part, like corrections, additions, etc. Each forthcoming (open and closed) data set reflecting the actual content of the data base after inclusion of new studies or substantial modifications due to new insights will be identified by a release number including the year it was issued.

2. Rationale

2.1 Statistical inference

Statistical conclusions are based on data sets. These are assumed to be representative of an underlying population with its distribution of variates. This inductive inference can never be absolutely reliable. On the contrary: Errors of inference are unavoidable. Under favourable conditions, however, the probability of an erroneous conclusion can be kept under control, as when for example it is stated that an estimated parameter lies in a certain interval with a probability of 95% or that a correct statistical hypothesis is, erroneously, rejected only with a probability of 5%.

Probability statements of this type are based on statistical models. Models describe the relationship between variables in general terms like 'proportional', 'linear', 'additive', 'independent', 'normally distributed' and so on. The probability statements about statistical conclusions are based on these model assumptions and therefore they are only (approximately) correct if the model assumptions are (approximately) satisfied.

2.2 Descriptive data analysis and generation of hypotheses

Statistical data analysis usually starts with a description of the data. Frequently various subgroups, such as male-female or older-younger patients are compared with each other on a purely descriptive basis. Such comparisons, however, lead to insights which may generate statistical hypotheses. E. g. if a difference in the distribution of a relevant parameter between older and younger patients is observed, this may easily lead to the formulation of the hypothesis, that there does not exist such a difference, in the hope of being able to show, that the observed difference is indeed significant. From the point of view of statistical theory, all hypotheses to be tested should be formulated before the start, or at least independently, of the descriptive analysis of the data. But in practice the description of the data frequently provides insights into the data set which lead to new questions and to a need to investigate the relationship by formulating hypotheses and by testing them formally.

2.3 Investigating model assumptions

A similar situation to the one described above also exists with respect to model assumptions. A scatter-plot or a suitable table can exhibit quite well whether a relationship between variables exists and whether this relationship is fairly linear or possibly quadratic or even exponential. The same holds for the question as to whether a parameter exhibits a symmetric or a highly skewed distribution, whether the variability in two subgroups is about the same and so on. All these aspects are very relevant for the formulation of a realistic model of the data within which formal statistical inferences will be drawn. Thus a thorough descriptive analysis of the data will lead to a more realistic model which, as mentioned above, is the basis for a trustworthy statistical conclusion.

2.4 Dangers of over-fitting and data-driven hypotheses testing

Although descriptive statistical analyses frequently lead to new insights into the data body and should therefore be highly recommended, they nevertheless present a substantial danger to the validity of formal statistical inferences (hypotheses testing and confidence sets) by destroying the probabilistic basis of inferential statistics. This can be seen as follows:

Every data set exhibits some features which pertain to the underlying population from which it has been drawn as well as some features which are purely accidental or random. If one were to collect a new data set on the same phenomenon, this data set would show the same population characteristics as the first one, but the random part will in general be quite different. The problem is that these two aspects of the data set cannot be differentiated and

therefore a purely incidental component of the data set may lead to the formulation of a scientific hypothesis. If, for example, by pure chance a difference between male and female patients is observed where previously none had been expected, this may lead to a formal test of this difference and possibly to a rejection of the hypothesis of equality, because this particular data set does support this hypothesis to some degree.

Thus a random feature of the data set leads to the generation and formulation of a related null hypothesis which has a good chance of being rejected mainly because of this random feature. Statistical theory guarantees that correct hypotheses are falsly rejected only in about 5% of all cases, but this theory assumes that the hypothesis has been formulated before, or at least independently of,any analysis of the data. Many theoretical as well as empirical investigations have shown that the probability of erroneously rejecting hypotheses generated by descriptive data analyses is usually much higher than the formally stated 5% and no upper limit for this probability can be given. Thus the major advantage of the statistical test, namely providing a bound on the probability of reaching a false conclusion, is lost. Many subject-area researchers are not aware of this fact and state the contrary in their publications by refering to the 5% probability of an erroneous conclusion.

Similar arguments can be applied to the situation of using the data set for modelling the data. This also leads to invalidating the probability basis of the statistical inference. However, in this situation the effect in general is not as substantial as in the case of data-driven generation of hypotheses.

It is important to note that the risk of over-fitting increases when the number of modelling parameters increases. Therefore caution should be taken to set a realistic limit to the ratio of number of parameters to sample size and not to draw far-reaching conclusions from data containing few patients but many variables.

2.5 Interaction in model checking

Valid statistical conclusions can only be drawn on the basis of a sound statistical model. Unfortunately it is difficult to check the model assumptions (independence, distribution, functional relationship, etc.) in a logically consistent fashion. Typically one of the model features can be checked only if one assumes that all the other relevant assumptions are satisfied.

For example, in a regression situation the question of a normal distribution of the errors can only be approached on the basis of the residuals as estimates of the errors. But the residuals can only be computed when the type of the relationship (linear, quadratic, exponential, etc.) is known and the relevant parameters have been estimated. However, the method of estimation of parameters in turn may depend on the error distribution. Thus there is a circular interaction between various aspects of model verification. This problem gets even more complicated when the assumption of independence also has to be assessed.

All these model checking steps, which have to be carried out before substantive inferences can be drawn from the data, will suffer if a purely random feature of the sample conveys a misleading picture. Therefore, some authors have proposed to check different aspects of the model assumptions on different subsets of the database and, in addition, perform an overall

validation test. In the context of prediction Hastie et al. (2001) make the following statement:

"If we are in a data-rich situation, the best approach for both model assessment and model selection is to randomly divide the dataset into three parts: (i) a training set, (ii) a validation set, and (iii) a test set. The training set is used to fit the models; the validation set is used to estimate prediction error for model selection; the test set is used for assessment of the generalization error of the final chosen model…".

Similarly, Van Houwelingen and le Cessie (1990) put their recommendations into these terms:

"Ideally, the data set should be split into three parts: one to select covariates, a second to estimate the regression coefficients and a third to assess the prediction rule”.

Hence, for the prediction context, these authors recommend splitting the sample into three or more parts, depending on the complexity of the model structure, but they qualify their recommendations by restricting it to an ideal, 'data-rich' situation.

Such recommendations are well justified if, in fact, the data base is extremely large, as might be the case in some technical fields, where hundreds of thousands of observations are available. In such situations the data bases, even after splitting into more than two parts, remain large enough to provide sufficient power for statistical tests. The advantage of a large data set is that even slight deviations from a statistical hypothesis will be detected with high probability. On the other hand, in a small data set even a medically significant difference (e. g. between two treatments) can remain undetected because of adverse random fluctuation ('false negative'). In medical research with a few hundred or possibly a few thousand patients the danger of losing power by splitting a data base into several small parts cannot be compensated by refining the model assumptions. In particular, the data base of the SLCMSR is not sufficient in size to allow for the corresponding dilution of statistical power. We therefore recommend to split the data into only two subsets.

2.6 Splitting the data set

Splitting the data-base randomly and using one part for model formulation and generation of hypotheses and using the remaining part solely for estimation of model parameters and testing hypotheses is the safest way to avoid the above-mentioned sources of bias. The first part is then called the 'learning sample' or 'training sample', whereas the second part acts as the 'validation sample' or 'confirmation sample'. This method was proposed in the context of discriminant analysis a long time ago, since it was observed that computing the discriminant function from a data set and then using this function for the same data set underestimates the error rates of discrimination substantially if the data set is small and the number of variables is large in comparison (see Fisher 1936).

The disadvantage of this method is that less than the full sample size is available for training as well as for validation. This loss of sample size decreases the precision of parameter estimates. It also decreases the power of rejection of a false hypothesis. If the class of hypotheses to be tested on the basis of the SLCMSR data is generated exclusively on medical grounds and if there is little doubt about proper modelling of the data, then such

a split is not justified. But then essentially no conclusion based on observed features of the data, beyond what was formulated beforehand, can be statistically justified.

On the other hand, using a part of the data for learning and requiring confirmation of the findings on the basis of the remaining part of the data along strict principles allows for tremendous freedom (see “Anything goes” Feyerabend 1993) of visualizing the data, searching for unexpected relationships and checking the model assumptions without loosing the probabilistic justification for the results finally arrived at on the basis of the validation sample. Under these circumstances looking at the training data from various angles is not only permitted but highly recommended.

On more theoretical grounds the following argument might provide some insight: There is a law of diminishing returns of the sample size in statistical investigations. Standard errors and the corresponding lengths of confidence intervals decrease not in proportion to n, but only in proportion to the square root of n. For example, doubling the sample size does not reduce the standard error to 50% of its previous value, but only to 71%. Thus the contribution ofeach additional observation decreases as the sample size increases. In this framework using half of the sample for training does not reduce the sensitivity of the remaining part to 50%, but only to 71% of the full sample. In this sense, the expense of 'wasting' about half the sample for learning is not as high as it might at first seem.

The fraction to be used for 'learning' has been discussed by many authors. We believe that since the validation is the essential part of the endeavour, at least 50% of the database should be reserved for this purpose. On the other hand, taking too few observations for the learning sample can lead to unreasonable models and unfruitful generation of hypotheses, because the purely random part of the learning sample will be predominant. Therefore, in the case of the SLCMSR data base, it seems the training sample should include at least 1/3 of the patients of the data base.

It is also important to note that the closed part of the data set is a finite resource. Every hypothesis which is tested using the closed part of the data set increases – slightly - the knowledge about the closed part and will therefore start to increase the likelihood of erroneous conclusions. Therefore, only hypotheses which are considered to have relevance should be subjected to validation.

2.7 Research risks and publication policy

Using the method of splitting the data base into a training sample and a confirmation sample in the case of the SLCMSR data base implies that the researchers will have at their disposal only the training sample. They will then have to use their experience and judgment to draw the greatest benefit from these data with respect to valid statistical conclusions while at the same time avoiding to be mislead by random aspects of the data.

A presentation of results, such as a publication or an oral presentation, should contain the information which findings on the open part have been confirmed when using the closed part. It does not seem to be ethical to present preliminary statistical results when, without substantial effort, a validation step can be performed. Thus, shortly before publication, the statistical methods applied to the training set up to that point will have to be applied to the confirmation set. It is the risk of the researcher that his preliminary findings might then not

be confirmed by the validation process. He can minimize this risk, however, by trying to avoid any degree of over-interpretation of his findings in the training sample.

The policy proposed here sets a standard for statistical investigations which considerably supersedes the care usually adopted in this respect. It is therefore recommended that this fact be stated explicitly when presenting results based on this restrictive policy. A statement along the following lines at the beginning of a publication/oral presentation can make this explicit:

"In providing data for statistical research, the SLCMSR has adopted the following policy for maintaining a high standard for statistical inference: The data base is split into an open and a closed part. The open part is made available to the researcher for exploration and investigation. The findings are then validated on the closed part of the database. This policy has been adopted in order to prevent the possibly substantial bias of exploring data and then, using the same dataset, performing formal statistical tests for hypotheses which have been suggested by such explorations. In the following, the results are those that have been obtained from the open part of the data base annotated by information about the validation of these in the closed part of the data base."

In order to protect the integrity of the validation data set, no data snooping should be allowed with the validation set. Rather, the researcher should be asked to hand over his methods and software to a group of trustees for the validation on the basis of the confirmation set.

3. Outline of a procedure to guarantee a high standard of statistical research on the basis of the SLCSMR data base

In order to implement these procedures for a statistical analysis of the SLCMSR data based on the strictest principles the following steps should be taken:

Researchers working in the field of statistical analysis of MS obtain only data from the learning sample. Only when their work is essentially ready for publication or for a presentation at a scientific meeting are they permitted and obliged to submit a request for confirmation, based on the validation sample to the Scientific Director of the Centre. After a positive check for feasibility, the request is forwarded to a validation committee consisting of the chairs of the SOC and the chairs of the MRI Working Group and the Clinical Research Working Group. After a positive check for medical relevance (turnaround time should not exceed 2 weeks), the request for validation is forwarded to the group of 'data trustees' which is responsible for the validation data set. This group guarantees that the validation data set is not used except for the confirmation of findings as explained above according to fixed rules (SOP's).

As the validation process temporarily interrupts the preparation of a publication, both preassessment of the request and statistical validation procedures must occur in a timely fashion. Strict adherence to this principle is of utmost importance for the scientific efficiency of the SLCMSR and collaborating researchers.

Whenever the SLCMSR data set gets enlarged by data from a new study, the trustees retain a fixed part, drawn at random from the new study data, and add it to the validation data base, while the rest is used to increase the open data set.

Since access to the closed part is controlled by the trustees and is limited substantially, learning more about its features beyond the results on parameter estimation and tests is essentially forestalled. Thus this part retains its virginal state and can therefore be used repeatedly. In particular, statistical results from a first round of investigations may lead to more sophisticated questions and models. If an analysis of this new approach on the basis of the learning sample seems promising, it again can be put to test using the closed part. As long as this follows the above rules it should not be construed as data snooping.

4. Acknowledgements

We thank the following colleagues for helpful comments and discussions:

B. Hellriegel, L. Kappos, S. Kessner, C. Lederer, S. Menzler, D. Miller, J. Petkau, M. Scholz, J. Wolinsky, P. Young and the members of the MRI working group

5. Literature/Further reading

Hans-Peter Beck-Bornholdt, Hans-Hermann Dubben, „Der Hund, der Eier legt. Erkennen von Fehlinformation durch Querdenken“, Imke Hoffmann (Herausgeber), Rowohlt Tb., 2001, ISBN: 3499611546

K.P. Brunham and D. R. Anderson, “Model Selection and Inference – A Practical Information-Theoretic Approach”, Springer, 1998

Paul Feyerabend, “Against Method”,. W W Norton & Co (Ssd) 1993, ISBN: 0860916464

Fisher, R.A. , "The Use of Multiple Measurements in Taxonomic Problems," Annals of Eugenics, 7, 179-188 (1936).

Thomas S. Kuhn, “The Structure of Scientific Revolutions”, University of Chicago Press, 1. November 1996, ISBN: 0226458083

Trevor Hastie, Robert Tibshirani, Jerome Friedman, “The Elements of Statistical Learning – Data Mining, Inference and Prediction”, Springer Series in Statistics, p 193 ff, 2001

van Houwelingen, J. C. and S. le Cessie, “Predictive Value of statistical Models”, Statistics in Medicine, 9, 1303-1325 (1990)

McCarthy, P. J. “The use of balanced half-sample replication in cross-validation studies”, Journal of the American Statistical Association, 44, 596-604 (1976).

Mosteller, F. and Tukey, J. W., Data Analysis and Linear Regression, Addison-Wesley, Reading, Mass., 1977
